# Revisiting the “jelly-roll” technique: New utility in diagnosing linear IgA bullous dermatosis mimicking toxic epidermal necrolysis

**DOI:** 10.1016/j.jdcr.2023.08.041

**Published:** 2023-09-14

**Authors:** Samavia Khan, Edward Bae, Regine J. Mathieu, Christopher Elco, Cathy M. Massoud, Elnaz F. Firoz

**Affiliations:** a Department of Dermatology, Rutgers Robert Wood Johnson Medical School, Somerset, New Jersey; b Department of Dermatology, Rhode Island Hospital, Warren Alpert Medical School of Brown University, Providence, Rhode Island; c Department of Pathology, Rhode Island Hospital, Warren Alpert Medical School of Brown University, Providence, Rhode Island

**Keywords:** drug eruptions, frozen sections, inpatient dermatology, linear IgA bullous dermatosis, vesiculobullous

## Introduction

Linear IgA bullous dermatosis (LABD) is an autoimmune-mediated condition with characteristic direct immunofluorescence (DIF) findings of linear IgA deposition along the basement membrane.^1^ This entity is typically idiopathic; however, it may also be induced by medications, most commonly vancomycin. LABD is characterized by tense bullae, yet some clinical cases may present with flaccid bullae, full-thickness desquamation, positive Nikolsky sign, and mucosal involvement necessitating consideration of Stevens-Johnson syndrome/toxic epidermal necrolysis (SJS/TEN), erythema multiforme, and staphylococcal scalded skin syndrome.^2^ In such cases, clinicopathologic correlation including DIF microscopy is imperative to exclude SJS/TEN. We present a case of a patient with tense and flaccid bullae, positive Nikolsky sign, and mucosal involvement in the setting of multiple new medications in which SJS/TEN was suspected. This case underscores the importance of the jelly-roll analysis in diagnostically challenging cases where clinical suspicion for LABD is high.

## Case report

**Fig 1:**
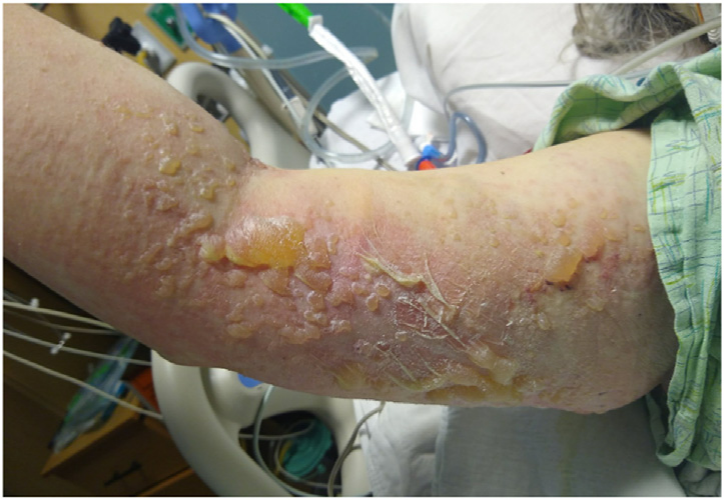
Right upper inner arm with both tense and flaccid bullae overlying erythema and full-thickness desquamation.

A 58-year-old woman with Graves disease, asthma, hypertension, hyperlipidemia, and seizures presented to the hospital in cardiac arrest after an episode of ventricular fibrillation. Her course was complicated by aspiration pneumonia for which she received intravenous piperacillin/tazobactam and vancomycin. Other medications administered included aspirin, atorvastatin, clopidogrel, pantoprazole, propofol, valproate, levetiracetam, and metoprolol. Five days into hospitalization, the primary team noted a rash; she continued receiving multiple antibiotics (ceftriaxone, amoxicillin/clavulanic acid, cefepime, vancomycin). Four days later, blisters on the skin prompted an inpatient dermatology consultation. Physical examination revealed admixed tense and flaccid bullae on the flank and upper extremities (Fig 1) with minor involvement of the proximal lower extremities covering 10% to 15% body surface area. Nikolsky sign was positive. Prebullous erythema involved the entire back. Mucosal exam revealed hemorrhagic crust of the distal tongue and erythema of the labia majora without full-thickness desquamation. Vital signs and laboratory findings were notable for a temperature of 101.3 °F and leukocytosis (white blood cell 25.4 × 10^9^/L) with eosinophilia (absolute count 1.3 K/μL).

**Fig 2:**
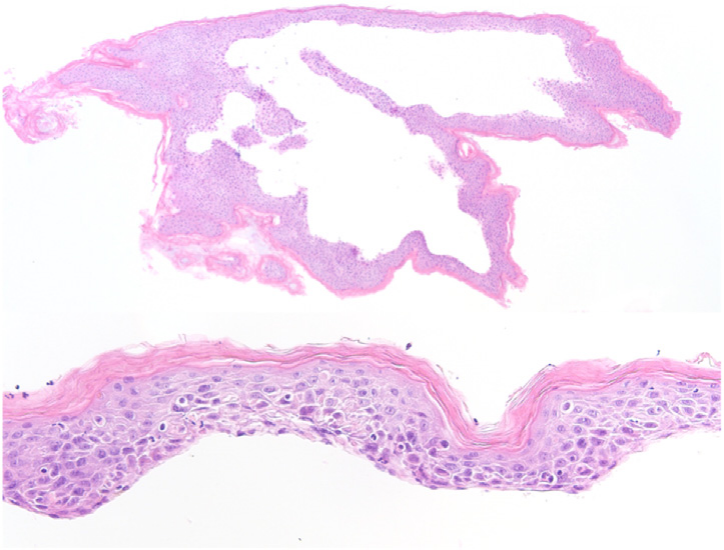
Jelly-roll section of detached skin from the roof of a bulla demonstrating orthokeratosis overlying full-thickness vital epidermis with spongiosis and scattered neutrophils (hematoxylin and eosin: 40× *top*; 400× *bottom*).

Several skin biopsies were obtained. Skin for fresh frozen analysis was obtained by employing the jelly-roll technique. Gentle back-and-forth motions with a cotton-tipped applicator were used to remove the sloughed skin from the roof of a flaccid bulla. This motion was effective in displacing adherent edges and no additional tools such as a scalpel or curved scissors were required. The sloughed skin was wrapped around the cotton-tipped applicator for handling, placed into a specimen jar containing normal saline, and then submitted to pathology for fresh frozen analysis. Given the need for rapid analysis of the fresh tissue, the pathology team in the hospital was notified prior to the procedure. Two punch biopsies (4 millimeter) of lesional and perilesional skin were obtained for hematoxylin and eosin analysis and DIF, respectively. Fresh frozen analysis showed a full-thickness epidermis with spongiosis and scattered inflammatory cells including neutrophils (Fig 2). The lack of full-thickness necrosis on jelly-roll analysis argued against SJS/TEN. Lesional punch biopsy revealed a subepidermal bulla with neutrophils on hematoxylin and eosin. Perilesional DIF demonstrated linear IgA deposition along the basement membrane, thereby supporting a diagnosis of LABD. In the setting of vancomycin administration, a diagnosis of drug-induced LABD was made. Vancomycin was held and supportive wound care was initiated. Three weeks after the discontinuation of vancomycin, the lesions resolved.

## Discussion

Traditionally, the jelly-roll technique has been used to differentiate staphylococcal scalded skin syndrome from TEN.^3^^,^^4^ Frozen-section analysis in staphylococcal scalded skin syndrome shows intraepidermal cleavage at the granular layer, while TEN shows full-thickness necrosis down to the basal layer. As frozen sections are critical for the rapid analysis of potential TEN when time is of the essence,^4^ they can be equally helpful in quickly diagnosing neutrophilic disorders with a positive Nikolsky sign, as this case demonstrates. The absence of necrotic keratinocytes in this case effectively ruled out TEN. Interestingly, initial DIF was falsely negative, possibly due to sampling error or tissue processing. The presence of neutrophils on frozen section greatly increased clinical suspicion for LABD, therefore DIF was repeated and revealed diagnostic features of LABD. More recently, the jelly-roll technique also has been used in diagnosing other cutaneous entities including epidermolytic hyperkeratosis.^5^ This case highlights the underappreciated though crucial role of frozen-section jelly-roll sampling in the rapid analysis of neutrophilic bullous disorders mimicking TEN. Moreover, when clinical suspicion for LABD is high, it may at times be necessary to repeat the DIF to exclude potential false negative findings.

## Conflicts of interest

None disclosed.
